# Exploring non-coding variants and evaluation of antisense oligonucleotides for splicing redirection in Usher syndrome

**DOI:** 10.1016/j.omtn.2024.102374

**Published:** 2024-10-28

**Authors:** Belén García-Bohórquez, Pilar Barberán-Martínez, Elena Aller, Teresa Jaijo, Pablo Mínguez, Cristina Rodilla, Lidia Fernández-Caballero, Fiona Blanco-Kelly, Carmen Ayuso, Alba Sanchis-Juan, Sanne Broekman, Erik de Vrieze, Erwin van Wijk, Gema García-García, José M. Millán

**Affiliations:** 1 Molecular, Cellular and Genomics Biomedicine, Health Research Institute La Fe, 46026 Valencia, Spain; 2 Center for Biomedical Network Research on Rare Diseases (CIBERER), Instituto de Salud Carlos III, 28029 Madrid, Spain; 3 Joint Unit CIPF-IIS La Fe Molecular, Cellular and Genomic Biomedicine, 46026 Valencia, Spain; 4 University and Polytechnic La Fe Hospital of Valencia, 46026 Valencia, Spain; 5 Department of Genetics & Genomics, Instituto de Investigación Sanitaria-Fundación Jiménez Díaz University Hospital, Universidad Autónoma de Madrid (IIS-FJD, UAM), 28040 Madrid, Spain; 6 Bioinformatics Unit, Instituto de Investigación Sanitaria-Fundación Jiménez Díaz University Hospital, Universidad Autónoma de Madrid (IIS-FJD, UAM), 28040 Madrid, Spain; 7 Center for Genomic Medicine, Massachusetts General Hospital, Boston, MA, USA; 8 Program in Medical and Population Genetics and Stanley Center for Psychiatric Research, Broad Institute of MIT and Harvard, Cambridge, MA, USA; 9 Department of Neurology, Massachusetts General Hospital and Harvard Medical School, Boston, MA, USA; 10 Department of Otorhinolaryngology, Radboud University Medical Center, 6525 GA Nijmegen, the Netherlands

**Keywords:** MT: Oligonucleotides: Therapies and Applications, Usher syndrome, USH2A, non-coding regions, deep-intronic, splicing, pseudoexon, minigene, antisense oligonucleotides

## Abstract

Exploring non-coding regions is increasingly gaining importance in the diagnosis of inherited retinal dystrophies. Deep-intronic variants causing aberrant splicing have been identified, prompting the development of antisense oligonucleotides (ASOs) to modulate splicing. We performed a screening of five previously described *USH2A* deep-intronic variants among *USH2A* monoallelic patients with Usher syndrome (USH) or isolated retinitis pigmentosa. Sequencing of entire *USH2A* or USH genes was then conducted in unresolved or newly monoallelic cases. The splicing impact of identified variants was assessed using minigene assays, and ASOs were designed to correct splicing. The screening allowed to diagnose 30.95% of the studied patients. The sequencing of USH genes revealed 16 new variants predicted to affect splicing, with four confirmed to affect splicing through minigene assays. Two of them were unreported deep-intronic variants and predicted to include a pseudoexon in the pre-mRNA, and the other two could alter a regulatory *cis*-element. ASOs designed for three *USH2A* deep-intronic variants successfully redirected splicing *in vitro*. Our study demonstrates the improvement in genetic characterization of IRDs when analyzing non-coding regions, highlighting that deep-intronic variants significantly contribute to *USH2A* pathogenicity. Furthermore, successful splicing modulation through ASOs highlights their therapeutic potential for patients carrying deep-intronic variants.

## Graphical abstract

**Figure:**
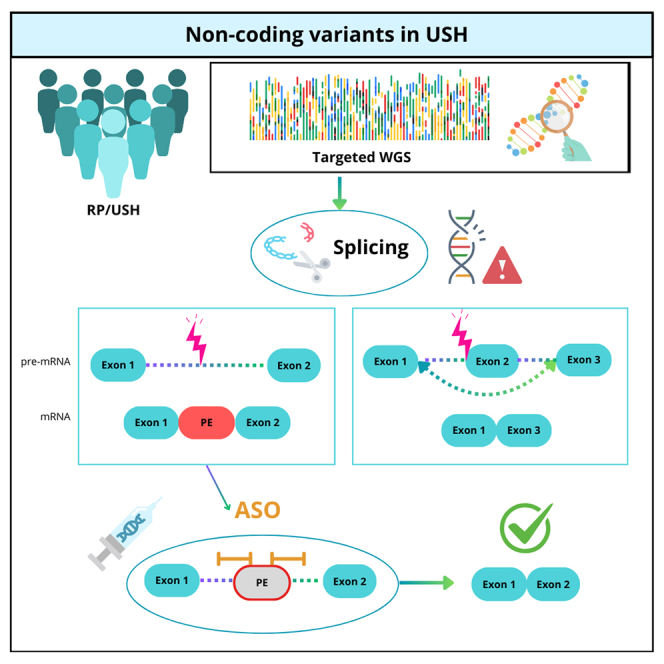

## Teaser

Non-coding regions are essential for IRD diagnosis. First, screening of five *USH2A* deep-intronic variants allowed to solve 30.95% of cases. Second, sequencing of entire *USH2A* or USH genes unraveled four variants with a validated effect in splicing. Eventually, ASOs successfully corrected splicing in three deep-intronic variants, highlighting their therapeutic potential.

## Introduction

Inherited retinal dystrophies (IRDs) are a group of rare disorders characterized by the progressive degeneration of the retina due to generally progressive photoreceptor or retinal pigment epithelium cell death. Although IRDs are considered rare diseases, they can account for 20%–25% of the blind working-age population, with 2 million people affected worldwide.^1^ IRDs are characterized by a high clinical and genetic heterogeneity, since pathogenic variants in 289 genes have been described to be causative (https://web.sph.uth.edu/RetNet/home.htm, accessed June 2, 2024), and each clinical entity can display different levels of progression and severity of the associated clinical symptoms. Within IRDs, retinitis pigmentosa (RP) is the most frequent IRD with a prevalence of 1/4,000 worldwide,^2^ and is caused by the initial degeneration of rods, followed by cone dysfunction and degeneration in advanced stages of the disease.

Besides being manifested as non-syndromic disorders, IRDs can also present as syndromic conditions, in which, besides visual impairment, also other clinical symptoms become apparent. Pathogenic variants in more than 200 different genes have been identified to be associated with syndromic forms of IRD.^3^ Particularly, RP can be associated with sensorineural hearing loss and balance impairments, known as Usher syndrome (USH). This syndrome explains more than 50% of hereditary deaf-blindness cases with a prevalence ranging from 4 to 17 individuals per 100,000.^4^^,^^5^^,^^6^ Typically, USH is classified into 3 types depending on the progression and severity of the symptoms and the age of onset. So far, pathogenic variants in 10 genes are known to underlie USH (*USH1C*, *MYO7A*, *PCDH15*, *CDH23*, *USH1G*, *CIB2*, *USH2A*, *ADGRV1*, *WHRN*, and *CLRN1*), although *CIB2* has been questioned as an USH-associated gene.^7^ Recently, a new USH clinical type, named USH4, has been defined and it is related to mutations in the *ARSG* gene.^8^^,^^9^^,^^10^^,^^11^^,^^12^^,^^13^ In addition, other genes have been reported to be associated in deafness-blindness syndromes similar to USH (*CEP78*, *CEP250*), as *USH2A*-phenotype modifiers (*PDZD7*), or result in a typical form of digenic inheritance with *ADGRV1* variants.^14^^,^^15^^,^^16^^,^^17^ Among all the USH genes, pathogenic variants in *USH2A* are the most prevalent, explaining 80%–90% of USH2 cases,^18^^,^^19^^,^^20^^,^^21^ and also the major cause of non-syndromic RP (nsRP) cases (RP39).^21^^,^^22^

Traditionally, the study of coding regions through a panel of genes or whole-exome sequencing (WES) has provided a diagnosis percentage of around 60% for IRD cases.^23^^,^^24^ Therefore, to achieve the complete characterization for those cases apparently monoallelic for pathogenic variants in recessive genes or not solved is a current goal in genetic diagnosis. In recent years, the analysis of non-coding regions of the genome has allowed to widen the range of variant types with a pathogenic effect that can be identified, thus emphasizing the importance of considering these regions for patient diagnosis.^25^^,^^26^^,^^27^

In this line, it is well known that deep-intronic variants are disease causing in IRDs.^28^^,^^29^^,^^30^^,^^31^^,^^32^ These variants promote the inclusion of a pseudoexon (PE) in the mRNA that leads to a truncated protein.^25^^,^^33^^,^^34^^,^^35^^,^^36^^,^^37^^,^^38^ Remarkably, the deep-intronic variant c.7595-2144A>G in *USH2A* has been reported to be recurrent as it may derive from a common ancestor, similar to the two most common variants in this gene, c.2276G>T and c.2299delG.^33^^,^^39^^,^^40^

Since many deep-intronic variants have been described as being pathogenic, antisense oligonucleotides (ASOs) have been introduced as promising future therapeutic molecules to correct aberrant pre-mRNA splicing.^25^^,^^40^ In addition, ASOs have been designed to induce the targeted in-frame skipping of (combinations of) frequently mutated exons.^41^^,^^42^ The first ASO, QR-421a/ultevursen, designed to induce the skipping of *USH2A* exon 13, has already reached the clinical phase with promising outcomes.

In this study, we performed a targeted screening of previously published deep-intronic *USH2A* variants in 42 clinically defined USH/nsRP patients, who were carriers of a single pathogenic variant in the *USH2A* gene. Subsequently, we studied the whole-genomic sequence of *USH2A* and/or all USH genes through a custom panel in monoallelic USH patients. Eventually, ASOs were designed to correct aberrant splicing caused by three identified deep-intronic variants that were shown to result in the inclusion of a PE. Assessment of the splicing redirection potential of the individual ASOs supported their therapeutic potential.

## Results

Screening of 5 reported deep-intronic variants by Sanger sequencing in 41 patients harboring a monoallelic *USH2A* variant completed the genetic diagnosis of 13 patients (30.95%) (Table 1). Variant c.7595-2144A>G was identified in 8 individuals (RP-1741 carried the c.9959-4159A>G and c.7595-2144A>G variants in heterozygosity). Variant c.14134-3169A>G was identified to be present in 3 patients and the variant c.9959-4159A>G was identified in 2 additional individuals.

**Table 1:** Reported *USH2A* deep-intronic variants detected by Sanger sequencing in the cohort

Patient	Family	Dx	Gene	Allele 1	Sanger screening	Reference
RP-331	–	ARRP	*USH2A*	c.2276G>T; p.(Cys759Phe)	c.7595-2144A>G	Dreyer et al.^43^
						Vaché et al.^33^
RP-654	FRP-30	USH	*USH2A*	c.11146C>T; p.(Gln3716∗)	c.7595-2144A>G	Vaché et al.^33^
RP-1647	FRP-417	ARRP	*USH2A*	c.2276G>T; p.(Cys759Phe)	c.7595-2144A>G	Dreyer et al.^43^
						Vaché et al.^33^
RP-1569	FRP-380	USH	*USH2A*	c.2299del; p.(Glu767Serfs∗21)	c.7595-2144A>G	Eudy et al.^44^
						Vaché et al.^33^
RP-1595	FRP-389	USH	*USH2A*	c.10636G>A; p.(Gly3546Arg)	c.7595-2144A>G	Vaché et al.^33^
RP-1596				c.10636G>A; p.(Gly3546Arg)	c.7595-2144A>G	Garcia-Garcia et al.^45^
RP-1776	FRP-484	ARRP + SNHL	*USH2A*	c.2276G>T; p.(Cys759Phe)	c.7595-2144A>G	Vaché et al.^33^
						Garcia-Garcia et al.^45^
RP-2055	FRP-619	USH	*USH2A*	c.8435_8438del; p.(Thr2812Metfs∗17)	c.7595-2144A>G	Aller et al.^46^
						Vaché et al.^33^
RP-1741#	FRP-460	USH	*USH2A*	c.7595-2144A>G; p.(Lys2532Thrfs∗56)	c.9959-4159A>G	Vaché et al.^33^
						Liquori et al.^34^
RP-1485	FRP-344	USH	*USH2A*	c.1214del; p.(Asn405Ilefs∗3)	c.9959-4159A>G	Bernal et al.^47^
						Liquori et al.^34^
RP-1472	FRP-341	USH	*USH2A*	c.2299del; p.(Glu767Serfs∗21)	c.14134-3169A>G	Eudy et al.^44^
						Baux et al.^35^
RP-1494	FRP-348	USH	*USH2A*	c.2299del; p.(Glu767Serfs∗21)	c.14134-3169A>G	Eudy et al.^44^
						Baux et al.^35^
RP-2224	FRP-722	USH	*USH2A*	c.2431_2432del; p.(Lys811Aspfs∗11)	c.14134-3169A>G	Nájera et al.^48^
						Baux et al.^35^

RP/RPN, patient number; FRP/FRPN, family number; Dx, diagnosis; ARRP, autosomal recessive retinitis pigmentosa; USH, Usher syndrome; SNHL, sensorineural hearing loss. “#” Refers to the NGS positive control.

Then, 27 patients with a monoallelic (likely) pathogenic variant in a USH gene were selected for targeted genome sequencing of all reported USH genes through a first custom panel (P1) containing the entire sequence of 13 USH genes and 2,000 flanking bases of their UTR sequences (Table S1). Subsequently, a second custom panel (P2) that included all the genomic sequence of *USH2A* gene was sequenced in 6 monoallelic *USH2A* patients (Table S1). The majority (97%) of the bases was sequenced with a depth coverage of ≥20× in both panel designs. Around 8,000 variants per patient were identified by P1 and 2,500 by P2. The sequencing of P1 allowed us to solve 3 cases and the genetic diagnosis was completed in a fourth case by the analysis of P2.

As a validation of the pipeline and filters applied for the analysis, and described in the materials and methods, the two previously detected deep-intronic variants c.9959-4159A>G and c.7595-2144A>G present in the RP-1741 case were correctly identified.

**Figure 1:**
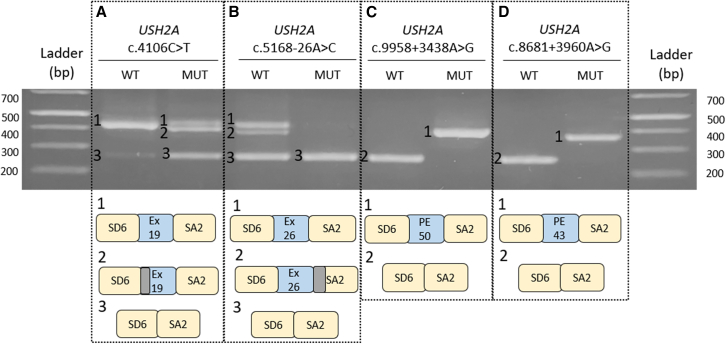
Analyses of potential splicing modulating variants in *USH2A* using minigene splice assays SD6 and SA2 are the constitutive exons of pSPL3 plasmid and in blue boxes exons (ex) and pseudoexons (PEs) are depicted. The first three variants present a(n) (partial) exon-skipping effect, while the last two have included a PE in their sequence. Boxes in gray represent regions that have not been included in the sequence as an alternative splicing has occurred.

From the 32 monoallelic analyzed cases, we selected a total of 16 variants with pathogenic scores according to splicing prediction tools and the cutoffs defined in materials and methods: 12 in *USH2A*, 1 in *CDH23*, 1 in *ADGRV1*, and 2 in *USH1C* (Table 2). Out of the 16 variants predicted to affect splicing, 11 deep-intronic variants, 2 intronic variants in close vicinity of an exon, and 4 exonic variants were identified (Table 2). The effect of the selected variants on splicing was studied using minigene splice assays and aberrant splicing was observed in 4 out of 16 variants (Table 2): 2 deep-intronic variants in *USH2A* (c.8681+3960A>G and c.9958+3438A>G), 1 intronic variant upstream of *USH2A* exon 26 (c.5168-26A>C), and 1 variant in *USH2A* exon 19 (c.4106C>T) (Figure 1). Among the 16 variants analyzed using minigene splice assays, variant c.1234G>A in the *USH1C* gene of patient RP-1222 was classified as a variant of unknown significance when it was identified in previous studies. Nonetheless, as a potential splicing effect was predicted by Splice AI (score: 0.31) in this study, we decided to select it for functional assay. However, the minigene analysis for this variant did not show any aberrant effect in splicing. All products shown in Figure 1 were confirmed by Sanger sequencing.

**Table 2:** Predictions for candidate variants potentially altering splicing

Patient	Gene	Variant identified	regSNP - intron	NNSplice		MaxEnt			SpliceAI	SPiP interp.	SPiP % (rank)	VarSEAK	Minigene effect
			Predict.	WT	MUT	WT	MUT	Var (%)	Score			Class	
**RP-2034**	***USH2A***	**c.5168-26A>C**	**B**	–	–	–	–	–	**0.55 (AL)**	**BPA**	**43.04 (35.2–51.14)**	**1**	**ES**
RP-1496	*ADGRV1*	c.3443G>A	–	–	–	–	–	–	0.53 (AG)	NEP	03.72 (01.51–07.52)	1	NE
**RP1950**	***USH2A***	**c.4106C>T**	**-**	**-**	**-**	**-**	**-**	**-**	**0.31 (AL)**	**REA**	**54.00 (45.68–62.16)**	**1**	**ES+NAS**
		c.11549-5dup	–	–	–	8.57	2.16	−74.8 (AL)	–	NEP	01.85 (00.38–05.32)	1	NE
RP-1222	*USH1C*	c.1234G>A	–	–	–	–	–	–	0.31 (AL)/0.34 (DL)	REA	85.91 (79.27–91.06)	1	NE
**RP-1036**	***USH2A***	**c.8681+3960A>G**	**PD**	**-**	**0.99 (AG)**	**1.09**	**9.84**	**802.75 (AG)**	**0.98 (AG)**	**NEP**	**00 (00–00.92)**	**5**	**PEC**
RP-1600	*USH2A*	c.15298-1252T>G	B	–	–	−1.63	6.97	527.61 (AG)	–	NEP	00 (00–00.92)	1	NE
RP-1943	*USH2A*	c.6049+3895G>A	B	–	0.92 (AG)	2.44	4.93	102.05 (AG)	–	NEP	00 (00–00.92)	1	NE
RP-1455	*USH2A*	c.5299-2503A>G	B	–	0.58 (AG)	−3.79	4.96	230.87 (AG)	–	NEP	00.25 (00.01–01.39)	1	NE
RP-1994	*USH2A*	c.11389+2566A>G	PD	–	0.73 (AG)	−0.93	7.82	940.86 (AG)	–	NEP	00 (00–00.92)	1	NE
		c.11048-2124A>G	PD	–	0.81 (AG)	−2.14	6.61	408.88 (AG)	–	NEP	00 (00–00.92)	4	NE
RP-2264	*USH2A*	c.7120+4268A>G	D	–	0.65 (DG)	−4.04	4.14	202.48 (DG)	–	NEP	00 (00–00.92)	1	NE
RP-2239	USH2A	c.7300+8957A>C	B	0.46	0.74(AG)	6.22	5.44	19.61 (AG)	–	NEP	00 (00–00.92)	1	NE
RP-2268	USH2A	c.6806-810A>G	D	–	0.92 (DG)	−0.37	7.81	2210.81 (DG)	–	NEP	00.5 (00.06–01.78)	3	NE
**RP-1815**	***USH2A***	**c.9958+3438A>G**	**PD**	**-**	**0.98 (AG)**	**1.66**	**10.41**	**527.11 (AG)**	**0.97 (AG)**	**NEP**	**00 (00–00.92)**	**3**	**PEC**
		c.4628-27169C>G	D	–	0.57 (DG)	−1.77	6.49	466.67 (DG)	–	NEP	00 (00–00.92)	2	NE

For regSNP-intron, NNSplice, and SpliceAI predictors, the scores are given within 0 (benign) to 1 (pathogenic). For VarSEAK the score ranks from 1 (benign) to 5 (pathogenic). Variants highlighted in bold showed an aberrant effect in splicing in the minigene assays. RP, patient number; NT, nucleotide; predict., prediction; B, benign; PD, probably damaging; TPR, true positive ratio; FPR, false positive ratio; WT, wild type; MUT, mutant; AG, acceptor gain; DG, donor gain; Var, variation; AL, acceptor loss; DL, donor loss; Interp., interpretation; BPA, branchpoint alteration; NEP, no effect predicted; REA, regulatory element alteration; ES, exon skipping; NE, no effect; NAS, new acceptor site; PEC, pseudoexon creation.

Concerning the copy-number variation (CNV) analysis, four rearrangements were identified (Table 3) and two of them were validated using a second technique: one 6.2 kb duplication in *USH2A* in patient RP-2159 by multiplex ligation-dependent probe amplification (MLPA) and one 2.5 kb duplication in *CDH23* in patient RP-681 by CytoScan HD array. The 2.4 kb deletion in *ADGRV1* and the 5 kb deletion in *MYO7A* in patients RP-1496 and RP-2213, respectively, turned out to be false positive as results from qPCR showed no alteration in the copy number.

In summary, after these combined studies, a total of 19 index cases were completely characterized, 18 of them having a previous pathogenic variant identified in *USH2A* and one in *CDH23*.

### *USH2A*: c.4106C>T

This study allowed us to re-evaluate the pathogenic effect for the variant c.4106C>T, identified in patient RP-1950 monoallelic for the c.2299del (p.Glu767Serfs∗21) variant. A score of 0.31 from SpliceAI predicted a splice acceptor loss, supported also by SPiP with a risk of 54% of altering an exonic splicing regulatory element (rank from 45.68% to 62.16%). Assessment of this variant in a minigene splice assay showed that this variant resulted in the (partial) skipping of exon 19 (Figure 1A). In Figure 1A, fragment 1 corresponds to the wild-type situation, whereas fragment 2 represents a partial skipping of exon 19 as the first 27 nucleotides are missing since an alternative splice acceptor/donor site was used. This event is in frame and no premature stop codon was predicted. Fragment 3 shows the complete skipping of exon 19, where only constitutive plasmid exons are included in the cDNA. This event is predicted to create a stop codon 17 nucleotides downstream of the variant and could generate a truncating protein. However, premature termination codons in USH genes are more likely to cause loss of transcripts due to nonsense-mediated decay. Fragment 1 corresponds to approximately 32.8% of total transcript count, while fragments 2 and 3 account for 67.2%. Segregation analysis demonstrated that variant c.4106C>T was in *trans* with mutation c.2299del, thus it co-segregates with the disease (Figure S1).

### *USH2A*: c.5168-26A>C

Variant c.5168-26A>C, which flanks exon 26 of *USH2A*, was identified in patient RP-2034. This patient presented a clinical USH2 diagnosis and was monoallelic for the *USH2A* c.2299del (p.Glu767Serfs∗21) variant. The prediction score from SpliceAI suggested a splice acceptor loss (0.55), while SpiP showed a potential branchpoint alteration with a score of 43.04% (35.2%–51.14%) (Table 2). To test the effect on splicing, a minigene construct was designed. The cDNA analysis showed a pathogenic effect for the mutant construct as a complete exon 26 skipping effect is observed (Figure 1B). In the wild-type construct, we observe three different bands, with bands 1 and 2 exclusively present in the wild-type context. Fragment 1 corresponds to the recognition of exon 26 and fragment 2 results from the lack of recognition of the first 43 bp of a pSPL3 constitutive exon (Figure 1B). Eventually, fragment 3 corresponds to the coding sequence from the pSPL3 constitutive exons, which means that exon 26 of *USH2A* is not recognized. The *in silico* analysis predicted a premature stop codon to be created 92 nucleotides downstream of exon 26 resulting in a truncating protein. This exon skipping event is present in both the wild-type and mutant constructs, but the exon skipping band accounts for 100% of the total transcript in the mutant minigene compared with 34.6% for the wild-type minigene. Segregation analysis confirmed that this variant is present in *trans* with the c.2299del and co-segregates with the disease (Figure S1).

**Table 3:** Copy-number variants identified

Patient	Family	Gene	Transcript	Genomic region	Location	Type	Validation method
RP-1496	FRP-350	*ADGRV1*	NM_032119	89909918–89912318	int1-int2	DEL	qPCR
RP-2213	FRP-719	*MYO7A*	NM_000260	76862211–76867161	int4-int5	DEL	qPCR
RP-681	FRP-49	*CDH23*	NM_052836	73335893–73360492	int8-int9	DUP	array HD
RP-2159	FRP-675	*USH2A*	NM_206933	215796232–216596790	int37-int43	DUP	MLPA

int, intron; DEL, deletion; DUP, duplication.

### *USH2A*: c.9958+3438A>G (PE50)

Variant c.9958+3438A>G was identified in patient RP-1815, who was diagnosed with USH2 and monoallelic for the c.908G>A (p.Arg303His) variant in the *USH2A* gene. The splicing predictor scores supported the variant pathogenicity as a result of an acceptor site gain (tgcAGtga). Alternative downstream donor sites were evaluated with NNSplice and SpliceAI 500, so we could identify a potential candidate donor site caagGTaatg (NNSplice: 0.98; SpliceAI 500: 0.85), which would lead to the inclusion of a PE of 144 bp. A fragment of 734 bp including the potential PE with ∼250 nucleotides of flanking up- and downstream intronic sequences was cloned into the pSPL3 plasmid. cDNA analysis after expression study showed a clear splicing effect where the predicted 144 bp PE was introduced in all of the detected transcripts, in the absence of any additional fragments (Figure 1C). Segregation analysis confirmed that both variants (c.9958+3438A>G and c.908G>A) are located on different alleles (Figure S1), thus supporting their pathogenicity as they co-segregate with the disease.

### *USH2A*: c.8681+3960A>G (PE43)

Deep-intronic variant c.8681+3960A>G was identified in patient RP-1036, who was clinically diagnosed with USH. RP-1036 harbored variant c.2809+1G>A in heterozygosity in *USH2A*. All splicing analysis tools revealed pathogenic scores (NNSplice: 0.99; MaxEnt: 802.75% variation; SpliceAI: 0.98; VarSeak: 5), and all of them concurred with an acceptor site gain effect. In addition, NNSplice and SpliceAI 500 tools were used to search for alternative donor sites that could promote the inclusion of a PE in the cDNA. Both predictors revealed the alternative donor site gaggGTaaga (NNSplice: 1.00; SpliceAI 500: 0.82), which was 111 nucleotides away from the new acceptor site. Considering this region as a possible PE, a minigene was designed including 738 bp of *USH2A* intron 43. Results from minigene assay revealed a full splicing effect in the mutant construct with the insertion of 112 intronic bp in the coding sequence (Figure 1D). The inclusion of this PE would promote the creation of a premature stop codon in the mRNA. DNA from relatives was not available for segregation analysis, so it remains unclear if the c.8681+3960A>G variant resides on the same allele as the previously identified canonical splice site variant c.2809+1G>A.

### ASO splicing redirection assay

The PE inclusion caused by the two deep-intronic variants identified in this study (c.9958+3438A>G and c.8681+3960A>G), and the previously described variant in intron 64 (c.14134-3169A>G), drove us to design ASOs with the aim of redirecting aberrant splicing, thereby preventing the PE inclusion in the coding sequence. Two ASOs were designed targeting the different PE regions, except for PE43 in which only one matched all requirements, and their efficacity was evaluated using minigene splice assays (Table S2). The two-nucleotide mismatch ASO (mmASO) used as control indicates the specificity of the ASOs as it is not able to redirect splicing.

**Figure 2:**
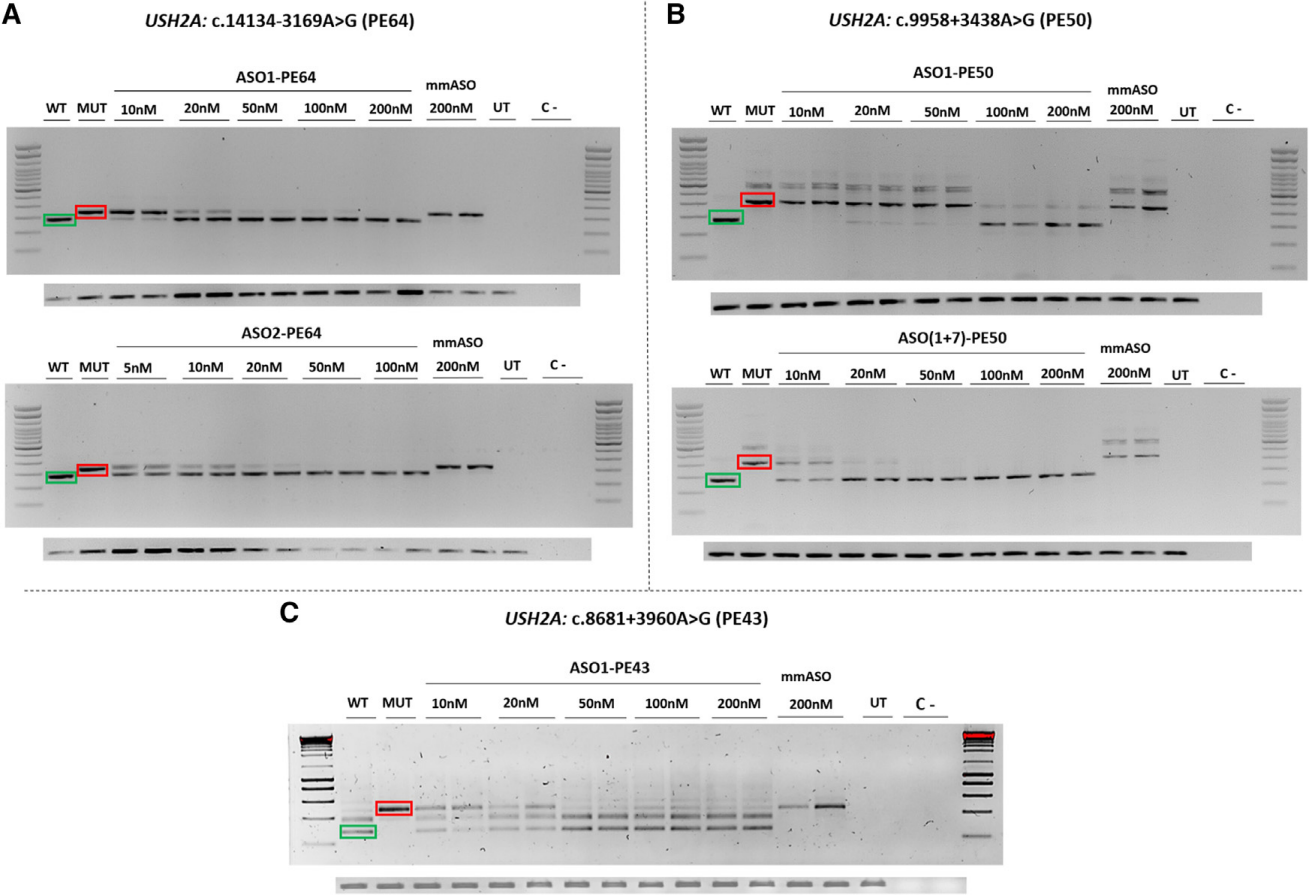
ASO-induced redirection of aberrant splicing caused by deep-intronic variants Each section represents WT (green) and MUT (red) fragments and the different treatment dose. The lower panels in every section are the GAPDH loading control in each experiment. For PE 64 (A), two different ASO were tested separately. For PE50 (B), ASO7-PE50 was tested together with ASO1-PE50 due to the lower efficiency it showed individually and the concentrations shown for ASO(1+7)-PE50 refer to the final concentration of both ASO. For PE43 (C), only ASO1-PE43 was tested as the sequence did not agree with all the requirements. Differences between wild-type and mutant fragments of PE50 and PE43 constructs in Figure 1 compared with this figure can be observed due to the use of different cloning splice vector for each experiment. WT, wild type; MUT, mutant; UT, untreated; C–, negative control.

Related to PE64, at a dose of 20 nM, ASO1-PE64 redirected the aberrant splicing, and it can also be observed how, from 10 to 20 nM, the fragment intensities show that the minimum efficient dose is 20 nM (Figure 2A). For ASO2-PE64, 5 nM is still effective for splicing redirection (Figure 2A). Concerning PE50, caused by c.9958+3438A>G, ASO1-PE50 was capable of redirecting the splicing at a dose of 100 nM (Figure 2B), while a combination of ASO1-PE50 and ASO7-PE50 was efficient enough at 20 nM (10 nM of each ASO), showing a synergic effect (Figure 2B). Results in PE43, due to c.8681+3960A>G variant, ASO1-PE43 showed great redirection of the splicing process with a minimum efficient dose of 50 nM (Figure 2C). The results obtained from the two mismatch ASOs used as a control (mmASO), showed a full specificity of all designed ASOs for each PE (Figure 2).

## Discussion

The diagnosis of both IRD and USH patients has commonly been accomplished by targeted NGS through custom panels and WES. Nevertheless, these approaches are focused on the coding sequence, which means that the role of regulatory and deep-intronic regions in the pathogenicity of these diseases is overlooked. Lately, more studies progressively emphasize the importance of non-coding regions when searching for pathogenic variants.^25^^,^^49^^,^^50^^,^^51^^,^^52^^,^^53^^,^^54^ This has already resulted in the identification of several deep-intronic variants in *USH2A* and *CLRN1* that result in the inclusion of a PE in the mRNA.^25^^,^^33^^,^^34^^,^^35^^,^^36^

After the study of our USH patients by either custom gene panels or WES, 10%–20% of analyzed individuals appeared to be monoallelic for a pathogenic variant in one of the USH genes in the absence of a second pathogenic variant.^55^^,^^56^^,^^57^^,^^58^^,^^59^^,^^60^ Motivated by this, we designed a custom panel including the whole genomic sequence of the USH genes to identify the second causative variant in non-coding regions and so unravel the genetic diagnosis in these cases.

It is remarkable how just the analysis of five deep-intronic previously reported variants in patients with monoallelic variants in the *USH2A* gene allowed us to complete the diagnosis in over 30% of the patients studied (Table 1). As expected, since it is one of the most frequent pathogenic *USH2A* variants found in the Mediterranean region, the intronic variant c.7595-2144A>G was also common in our cohort,^39^^,^^61^^,^^62^^,^^63^ found in 61.5% of the solved patients of the screening. Even more, we identified a patient carrying two different deep-intronic *USH2A* variants (c.7595-2144A>G and c.9959-4159A>G) both in heterozygosity, highlighting the importance of screening the non-coding regions, as classical approaches would have completely missed these variants.

The combination of whole USH genes sequencing along with *in silico* and *in vitro* studies, through minigene assays due to the low expression of USH genes in peripheral blood, allowed us to confirm aberrant splicing induced by four different variants.

On the one hand, it has allowed us to identify two new deep-intronic variants in two different patients and a PE inclusion was shown in minigene assays (c.8681+3960A>G and c.9958+3438A>G). On the other hand, an effect in cDNA was validated in two variants located in splicing regulatory elements of *USH2A* (c.5168-26A>C; c.4106C>T). Variant c.4106C>T in *USH2A* was described previously,^64^ but there was no evidence of its impact in splicing. In addition to the splicing predictions reported in this study, our minigene assay showed an exon skipping together with the creation of a new acceptor site. Nevertheless, the wild-type fragment is still present in the mutant context at 32.8%. Thus, we cannot confirm that this variant is clearly pathogenic as 20% of wild-type *USH2A* transcript has been reported to be enough for correct protein function.^41^ It should be taken into account that the outcomes observed by minigenes may differ from the *in vivo* situation since minigene assay is an *in vitro* model that presents some limitations. Minigene constructs incorporate only a part of the genomic sequence, which does not capture the full complexity of regulatory elements. Moreover, splicing can be different in different cell types due to the presence of cell-type-specific splice factors. In this line, complementary studies using patient-derived cells, such as photoreceptor precursor cells differentiated from patient-derived iPSCs, are crucial for obtaining a more accurate understanding of splicing events and their pathogenic consequences.^25^^,^^65^^,^^66^ Despite their limitations, minigene studies remain a very useful and practical tool for assessing potential splicing alterations in pathologies where the genes have low or no expression in accessible tissues.

Predicting the effect of nucleotide changes on splicing is challenging because this is a complex process regulated by numerous factors. Various studies have analyzed the sensitivity and specificity of different splicing predictors and cutoff values.^67^^,^^68^^,^^69^ Recently, Reurink and colleagues suggested to use a SpliceAI score of 0.1 for deep-intronic and canonical splice site variants, and a more restrictive cutoff of ≥0.2 for exonic changes in non-canonical positions.^25^ In this study, we selected variants with a score ≥0.2 for SpliceAI,^69^^,^^70^ and/or with a variation score >10% for MaxEnt.^34^ However, only 4 out of the 16 selected candidate variants (25%) in our study showed a deleterious effect on splicing, which led us to recommend a more stringent pipeline. Of the 4 variants whose splicing effect was functionally validated, all were predicted by SpliceAI. However, 2 of them would have been missed based solely on MaxEnt predictions. Among the 12 selected variants where an effect was not confirmed in the minigene assay, the majority were selected based on MaxEnt criteria. If we had only considered variants with a SpliceAI score ≥0.2, only 6 variants would have been selected for validation, and functional aberrant splicing effects would have been confirmed in 4 of them (67%). These data suggest that SpliceAI is consolidating itself as one of the best tools for predicting potential aberrant effects on splicing. In contrast, MaxEnt predictions alone yielded several false positives, so we would recommend using it in combination with other more accurate predictors.

Variants in UTRs have been already described in many genes with different effects on gene expression. However, predicting the effects of alterations in these regions remains challenging and cannot be done in a simple and automated way.^71^^,^^72^ In addition, functional assays are still necessary to assess the impact of these variants. Recently, Dueñas Rey et al.^73^ have developed a strategy for the prioritization and evaluation of 5′ UTR variants in combination with functional studies. They identified several variants in 5′ UTR predicted to have different functional consequences, highlighting the contribution of non-coding regions in IRDs. Considering the transcriptional complexity of the retina, more studies using ChiP-seq or RNA-seq techniques are needed for a better identification and definition of the regulatory regions.^74^^,^^75^^,^^76^^,^^77^

Several USH genes contain repetitive regions, which result in a lower capture efficiency. Therefore, we cannot rule out that variants in these poorly covered regions might have been missed due to technical limitations. Currently, a proper alternative would be whole-genome sequencing (WGS), as it helps to overcome these issues associated with sequence capture and provide more uniform sequencing.^25^^,^^78^ In a study carried out by Reurink et al. in 2023, 49% of patients who previously underwent WES analysis^25^ were solved by the identification of either a deep-intronic variant or SVs (structural variants) through WGS. This study also remarks on the relevance of SVs in IRD patients.^26^^,^^79^^,^^80^ In our study, four putative CNVs were identified using a specific bioinformatic pipeline, and only two of them were validated by different approaches. However, the complexity of some types of SVs makes their alterations difficult to detect using short-read sequencing technologies. To solve this limitation, long-read sequencing approaches or optical genome mapping are yielding promising results in the identification of SV and other complex alterations such as short repeat tandem.^81^^,^^82^

To redirect aberrant splicing caused by identified variants and prevent the inclusion of PEs in the coding sequence, ASOs were designed for the *USH2A* deep-intronic variants c.9958+3438A>G and c.8681+3960A>G identified in this study, as well as for the previously identified pathogenic *USH2A* variant c.14134-3169A>G.^35^ We obtained minimum efficient doses of 20 nM (ASO1-PE64) and 5 nM (ASO2-PE64) for c.14134-3169A>G; 100 nM (ASO1-PE50) and 20 nM (ASO(1+7)-PE50) for c.9958+3438A>G; 50 nM (ASO1-PE43) for c.8681+3960A>G. These results match previous data from deep-intronic variants in *USH2A*,^25^^,^^63^ and highlight the effectiveness of ASOs to modulate splicing through PE inclusion blocking. In total, every minimum efficient dose was below 200 nM, which was the maximum dose established for each experiment and which reassures their therapeutic potential.

ASOs have a high potential to modulate gene expression.^83^^,^^84^^,^^85^ Genetic approaches based on ASOs present certain advantages compared with other genetic intervention strategies such as gene augmentation: they can only temporarily interfere in the mRNA (so there is not the risk of permanent off-target effect at genomic level), endogenous gene expression levels are not modified and do not alter the transcript and protein isoform landscape of the target genes. Moreover, the small size of ASOs allows the intravitreal delivery with an effective reach of all retinal cells with low inflammatory effects instead of the use of AAVs, which requires surgical subretinal techniques for delivery and only a fraction of the retina will be reached resulting in a lower efficacy. Particularly for splicing correction, ASOs do not rely on preclinical animal testing, which is a requisite for exon-skipping approaches in which the resulting protein function should be proved, for instance, “ultervursen” for *USH2A* exon 13 skipping.^41^^,^^86^ This does not excuse the need for a dose finding study for ASOs modulating splicing, at least with an *in vitro* model such as 3D organoids. Also, toxicity assays are required (mostly in vertebrates, but sometimes in non-human primates).^87^ Furthermore, strategies improving the ASO targeting of specific tissues and cell types has also been described.^88^^,^^89^^,^^90^^,^^91^

To overcome the *in vivo* instability due to nuclease activity, in the second-generation ASOs some improvements have been done, such as including a phosphorothioate backbone and a sugar modification (2′-O-methoxyethyl [2′-O-MOE]).^92^^,^^93^ It has been reported that second-generation ASOs allow increased *in vivo* half-life times, being around 200 days.^86^^,^^94^ Despite this, some disadvantages involve the need for recurrent intravitreal injections. The use of AAV could be a good solution as its potential has been shown previously in cellular and animal models of Leber congenital amaurosis associated with a deep-intronic *CEP290* mutation.^95^ However, since viral vectors do not produce and deliver second-generation ASOs, the specificity and efficacy of the treatments would be affected. An alternative for this limitation is a strategy based on the enclosure of ASOs in a U7snRNA complex followed by an AAV-based delivery, which turned out to improve the long-term efficacy of the treatment.^96^

Regarding this, clinical trials based on the use of ASOs for treating IRD caused by a deep-intronic variant in *CEP290* as well as by variants harbored in exon 13 of *USH2A* were initiated (ClinicalTrials.gov: NCT03780257, NCT03140969, NCT03913143).^97^ However, after initially being discontinued by ProQR Therapeutics, Théa (https://www.laboratoires-thea.com) is currently preparing phase 3 clinical trials for both programs. In addition, an ASO-based allele-specific mRNA knockdown/degradation approach for dominantly inherited RP caused by pathogenic variants in *RHO* is underway in a clinical trial (NCT04123626), as are two different approved treatments for spinal muscular atrophy and Duchenne muscular dystrophy based on ASOs that modulate the splicing process.^98^^,^^99^ Although ASO-based splicing modulation is mostly a mutation-specific approach, mutational hotspots could also be a great target to focus on.

In summary, this study demonstrates the importance of the analysis of non-coding regions to solve some of the cases that are not diagnosed after gene panels, clinical exome, or WES. The molecular diagnosis is essential for the eligibility of receiving a gene- or mutation-based treatment. To date, between 30% and 40% of patients still do not receive a conclusive genetic diagnosis, which in part could be explained by the presence of pathogenic variants in regions that are (technically) difficult to sequence (for instance, ORF15 in *RPGR*). However, the presence of variants in non-coding regions (introns or *cis*-regulatory elements), the insertion of mobile elements and even epigenetic modifications could underlie the lack of diagnosis in the major part of these unexplained cases. Identification of (deep-)intronic variants that affect pre-mRNA splicing opens up future treatment options using antisense technology.

## Materials and methods

### Patient selection

Fifty-nine patients from 58 different families (Table S1) harboring a pathogenic variant in a USH gene and in whom the coding regions of the genes of interest had been previously studied, were selected (B.G.-B., G.G.-G., and J.M.M., unpublished data).^55^^,^^56^^,^^57^^,^^58^^,^^60^ Their diagnosis varied from any of the USH subtypes (42/59) to autosomal recessive nsRP (16/59) (Table S1). DNA from peripheral blood was isolated from all of them by automatic extraction with QIAsymphony (QIAGEN).

Different ophthalmologic tests were carried out in each patient, such as OCT, eye fundus, ERG, and evoked potentials. The Hospital La Fe Ethics and Fundacion Jimenez Diaz-University Hospital Ethics Committees approved this study in agreement with the Declaration of Helsinki. Informed consent was signed by all patients and relatives who participated in the study.

### Sanger screening of *USH2A* deep-intronic variants

We performed a Sanger screening of the 5 deep-intronic variants (c.7595-2144A>G; c.5573-834A>G; c.8845+628C>T; c.9959-4159A>G; c.14134-3169A>G) that were already described as pathogenic in *USH2A* gene in 41 carriers of 1 heterozygous mutation in this gene.^33^^,^^34^^,^^35^ We did not accomplish the screening in patients RP-2264 and RPN-803, who were previously sequenced with a custom panel containing these 5 variants. The 5 regions were analyzed as described previously.^33^^,^^34^^,^^35^

### Panels design and high-throughput sequencing

A first custom panel (P1) was designed including the whole-genomic sequence of the 13 USH genes and 2,000 flanking bases of UTRs of all of them (*MYO7A*, *CDH23*, *PCDH15*, *USH1G*, *USH1C*, *CIB2*, *USH2A*, *ADGRV1*, *WHRN*, *CLRN1*, *HARS*, *CEP250*, *PDZD7*)*.* The panel size encompasses 3,295.139 kb. The library preparation was performed following the Kapa HyperPlus Workflow (Roche) and it was sequenced in a NextSeq platform (Illumina, San Diego, CA) in 300 cycles of 2 × 150 bp reads. The P1 was sequenced in 27 patients, of which 17 had a mutation in *USH2A*, 3 in *ADGRV1*, 2 in *CDH23*, 2 in *MYO7A*, 2 in *USH1C*, and 1 in *PCDH15* (Table S1). Patient RP-1741 was included as an internal control for panel sequencing. This case carried 2 heterozygous deep-intronic variants in *USH2A* gene (c.9959-4159A>G and c.14134-3169A>G).

A second panel (P2) was designed including the whole-genome sequence of *USH2A*, since all remaining USH patients harbored one mutation in *USH2A*. Thus, a total of 779.928 kb was sequenced in six patients. The library was prepared following the SureSelectXT Target Enrichment System (Agilent Technologies) and sequenced in a MiSeq platform (Illumina) in 600 cycles of 2 × 300 bp. The P2 was sequenced in six patients, all of them carrying a mutation in *USH2A* gene (Table S1).

### Sequencing data analysis

Quality control of reads was performed using FastQC (v.0.11.4, https://github.com/s-andrews/FastQC) and samtools (v.1.3.1).^100^ The reads were aligned against the GRCh38 human genome of reference using BWA aligner (v.0.7.15-r1140),^101^ and single-nucleotide variants (SNVs) and small insertions and deletions (indels) were called using strelka (v.2.9.10).^102^ Variants were annotated with Variant Effect Predictor (ensembl-vep v.99.2),^103^ using MaxEnt and SpliceAI (v.1-3, https://github.com/Illumina/SpliceAI).

For variant filtering, we selected variants with a minor allele frequency of ≤0.01 and those located in the target gene. Subsequently, variants with either a SpliceAI score ≥0.2 or a MaxEnt variation score >10% and/or a value score ≥3 were used for acceptor/donor gain predictions. Additional *in silico* analysis was performed using the splicing predictors NNSplice (https://www.fruitfly.org/seq_tools/splice.html), VarSeak (https://varseak.bio/), regSNP-intron (https://regsnps-intron.ccbb.iupui.edu/), and SPiP (available in https://mobidetails.iurc.montp.inserm.fr/MD/) to predict alterations of natural splice sites. Variants predicted to create a new splicing site were further investigated using SpliceAI 500 (available in https://mobidetails.iurc.montp.inserm.fr/MD/) to trace the presence of alternative splicing sites, which can promote a PE inclusion. Variants supposed to alter either a branchpoint or an enhancer motif were studied with different predictors such as ESEfinder (http://krainer01.cshl.edu/cgi-bin/tools/ESE3/esefinder.cgi?process=home), SVM-BPfinder (http://regulatorygenomics.upf.edu/Software/SVM_BP/), and LaBranchoR (available in https://genome.ucsc.edu/). Alternative open reading frames caused by premature stop codons were analyzed for every variant in which an alteration in splicing was predicted (https://www.bioinformatics.org/sms/orf_find.html).

In addition, all coding regions were re-analyzed following the filters already described in previous studies.^55^^,^^58^

### CNV analysis

Analysis of CNV has been assessed using a bioinformatic pipeline (https://github.com/TBLabFJD/NextVariantFJD) including three different CNV detection programs: CoNVaDING,^104^ ExomeDepth,^105^ and panelcn.MOPS.^106^ Programs were run using a bed file defining windows of 150 bp within the captured regions^104^ to facilitate the performance of the algorithms, all based on sequenced depth comparison. Analysis batches were defined by gene panels and sequencing runs to avoid different coverages due to technical reasons. Variants were annotated using AnnotSV.^107^ We prioritized variants for further validation based on their presence in genes of interest, pathogenicity predictions, and number of programs that detected them. In addition, for cases with no variants identified the analysis was extended to the rest of genes.

Once variants were prioritized, they were validated by different techniques relying on the region to be analyzed: MLPA (MRC Holland; probemixes P361 and P362 for *USH2A*), CytoScan HD Array (Thermo Fischer Scientific), or qPCR.

### Validation and segregation of candidate variants

Validation of candidate SNVs was assessed by Sanger sequencing as described previously.^58^ Similarly, segregation analysis was performed in 4 families out of the 21 solved patients (Figure S1) since DNA from all relatives was not available.

### Functional validation of the identified deep-intronic variants

Splicing effects were validated by minigene assays. DNA was amplified by the Fidelity Phusion polymerase (Thermo Fisher Scientific. Waltham, MA). The products were purified and digested with either XhoI or NdeI and NheI. Afterward, digested products were cloned between restriction sites in pSPL3 plasmid (Dr. I. Botillo and Dr. S. Tuffery-Giraud), by using T4 ligase. The different primers of the regions amplified and cloned as well as the different minigene construct design are detailed in the supplemental information (Table S3; Figure S2). Transformation of *Escherichia coli* occurred by electroporation. Later, 1 μg of each clone was transfected in duplicate into HEK293 cells (Lipofectamine 3000 reagents, Thermo Fisher Scientific) grown in a 6-well plate. After 24 h, RNA was isolated using an RNeasy mini kit and reverse transcription was assessed using the PrimeScript RT Reagent Kit (TaKaRa, Kusatsu, Japan). Constitutive exons primers from pSPL3 were used to amplify the final product in a 2% agarose gel,^108^ from which the different bands were purified using a QIAquick Gel Extraction Kit (QIAGEN, Hilden, Germany). Eventually, the products were sequenced by Sanger and analyzed using the chromatogram viewer FinchTV (https://digitalworldbiology.com/FinchTV) to identify any modification due to an alteration in the splicing process. Calculating the transcript counts resulting from minigene assays was performed using ImageJ (https://imagej.net/ij/), an image processing program. First, we determined the intensity of the fragment of interest in relation to the background and then we calculated the relative intensity of this fragment with respect to the rest of the bands observed.

To test the splicing redirection potential of several ASOs, minigene constructions were built again as described previously.^109^ The pCI-neo plasmid, which contains exons of *RHO* allowing the study of splicing, was cloned using the gateway cloning approach. In this line, ASO assay could also match described guidelines and efficiency observed in recent work.^25^^,^^110^^,^^111^

### ASO design and testing

**Figure 3:**
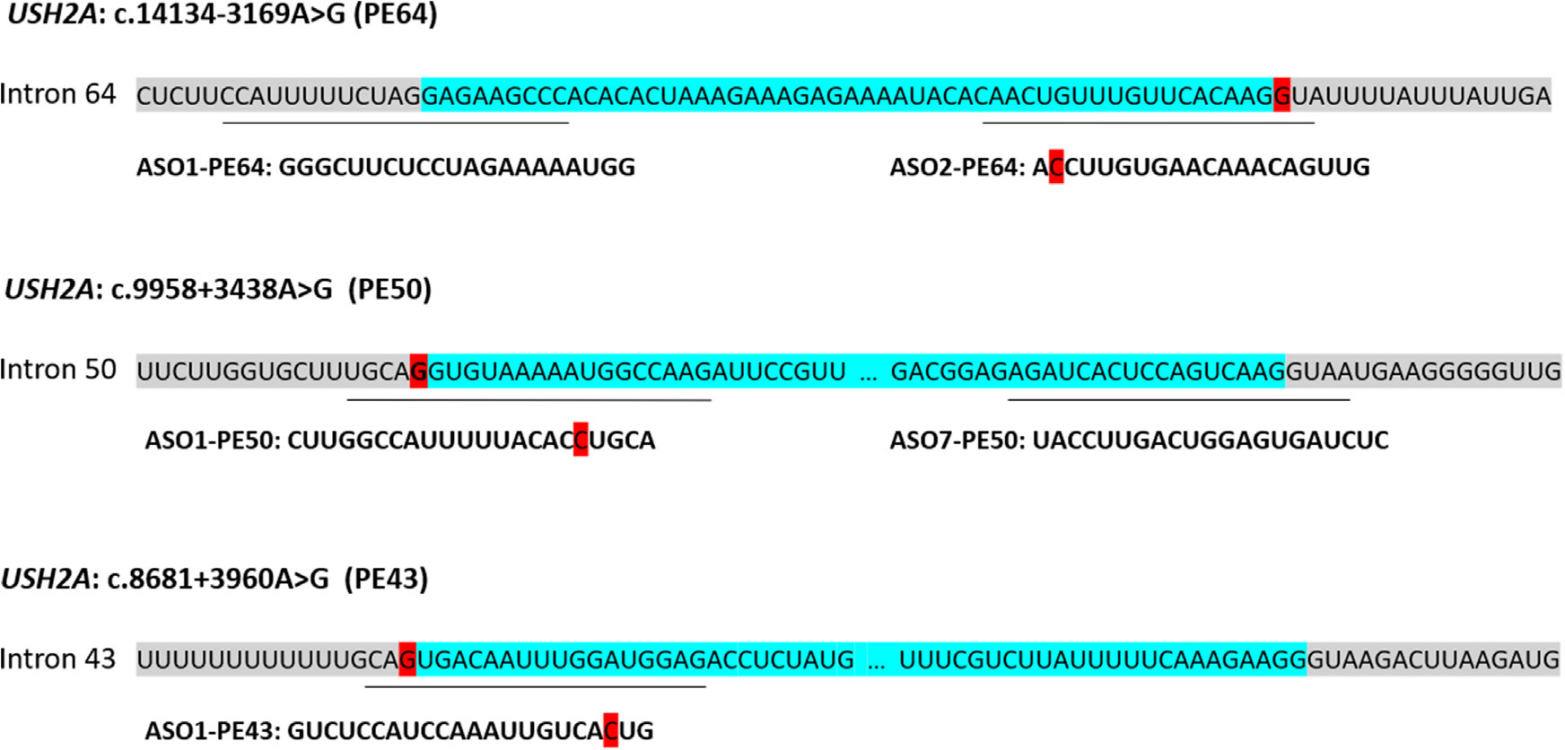
Position of the designed ASOs with respect to the different PEs Sequences in gray represent the intronic region, whereas the blue nucleotides correspond to the PEs. Variants are depicted in red. The lines below the different intron indicate the specific sequence for each ASO, which are also empathized right under them. PE50 and PE43 are disrupted by dots to make all sequences equal in length.

As mentioned in the section above, the designing of the ASOs was carried out following published guidelines,^110^^,^^111^ and purchased through Eurogentec (Seraing, Belgium). The ASOs were ordered with 2′-O-MOE modifications and a fully phosphorothiorate backbone. Two different ASOs overlapping the 5′ and 3ʹ boundaries between PE and intron were designed for PE50 (ASO1-PE50; ASO7-PE50) and PE64 (ASO1-PE64; ASO2-PE64), except for PE43 (ASO1-PE43), whose secondary structure made it complicated to target more than one region. For PE50 and PE64, ASOs were tested both separately and together by adjusting the final concentration to see if there was a possible synergic effect. The relative position of each ASO on the different PE sequence is depicted in Figure 3.

To ensure a specific binding of the ASO, unspecific targets were searched in NCBI blast. The ASO reconstitution was done in PBS 1× to a concentration of 1 mM and later aliquoted and diluted with PBS 1× to a concentration of 0.1 mM. Different volumes were co-transfected in duplicate with the pCI-neo-based minigene constructs with FuGENE HD transfection reagent (Promega) to test different doses of ASOs. Twenty-four hours later, cells were harvested and the RNA was analyzed. The ASOs were firstly tested at 20 and 200 nM both separately and together (for PE50 and PE64), which led to further studies in which the minimum effective doses were established. A 2 nucleotide mmASO was used as control for every experiment and it was transfected at 200 nM and in duplicate. Since it is not able to redirect splicing, this control ASO is an indicator of the specificity of the ASOs for each PE.

## Data and code availability

Genomic data will be available upon request. In addition, all sequencing data are deposited to a public repository under the dataset code EGAD50000000687 (European Genome-Phenome Archive).

## Acknowledgments

J.M.M. received two grants from 10.13039/501100004587Instituto de Salud Carlos III (ISCIII), “PI22/00213” and “AC21_2/00022.” G.G.-G. acknowledges two grants from 10.13039/501100004587Instituto de Salud Carlos III (ISCIII), “CP22/00028” and “PI22/01371,” co-funded by the 10.13039/501100000780European Union. G.G.-G. has also a grant funded by the European Union in the HORIZON programme HORIZON-HLTH-2023-TOOL-05-04 (BETTER, 101136262). P.B.-M. received a grant from 10.13039/501100023561Ministerio de Universidades “FPU20/04736”, co-funded by the 10.13039/501100000780European Union. This study was also supported by Stichting Ushersyndroom, de Gelderse Blindenstichting, and the 10.13039/100001116Foundation Fighting Blindness USA (grant no. PPA-0517-0717-RAD to E.V.W.). C.A. received a grant from 10.13039/501100004587Instituto de Salud Carlos III (ISCIII), “PI22/00321.”

## Author contributions

Conceptualization and supervision, J.M.M., G.G.-G., E.V.W., and E.d.V.; methodology, B.G.-B., P.B.-M., S.B., and E.d.V.; resources, B.G.-B., P.B.-M., E.A., C.R., L.F.-C., S.B., and E.d.V.; bioinformatic analysis and data curation, P.M. and A.S.-J.; writing – original draft, B.G.-B.; writing – review & editing, B.G.-B., P.M., A.S.-J., C.A., E.V.W., G.G.-G., and J.M.M.; funding acquisition, J.M.M. and G.G.-G. All authors have read and agreed to the published version of the manuscript.

## Declaration of interests

The authors declare no competing interests.

## Supplemental information

Document S1. Figures S1 and S2 and Tables S1–S3

Document S2. Article plus supplemental information
