# Supplementary material for: Exploring non-coding variants and evaluation of antisense oligonucleotides for splicing redirection in Usher syndrome
Source: Mol Ther Nucleic Acids. 2024 Oct 28;35(4):102374. doi: 10.1016/j.omtn.2024.102374 (PMC11612772; doi:10.1016/j.omtn.2024.102374)
Supplement: Document S1. Figures S1 and S2 and Tables S1–S3 [file mmc1.pdf]

OMTN, Volume *35*

Supplemental information

Exploring non-coding variants and evaluation of antisense oligonucleotides for splicing redirection in Usher syndrome

Belén García-Bohórquez, Pilar Barberán-Martínez, Elena Aller, Teresa Jaijo, Pablo Mínguez, Cristina Rodilla, Lidia Fernández-Caballero, Fiona Blanco-Kelly, Carmen Ayuso, Alba Sanchis-Juan, Sanne Broekman, Erik de Vrieze, Erwin van Wijk, Gema García-García, and José M. Millán

Table S1. Studies performed in the selected patients.

Patient	Family	Gene	Dx	Allele 1	SS	NGS	SEG	Reference
**RP-331**	**-**	***USH2A***	**ARRP**	**c.2276G>T; p.(Cys759Phe)**	**SP**	**NP**	**NA**	[1]
RPN-645	FRPN-243	*USH2A*<br>*CEP290*	ARRP	c.2276G>T ; p.(Cys759Phe)<br>c.7394_7395del; p.(Glu2465Valfs*2)	SP	P1	NA	[1]<br>[2]
**RP-654**	**FRP-30**	***USH2A***	**USH**	**c.11146C>T; p.(Gln3716*)**	**SP**	**NP**	**NA**	[3]
RP-681	FRP-49	*CDH23*	USH	c.7279del; p.(Thr2427Leufs*47)	NP	P1	NA	[4]
RPN-803	FRPN-372	*USH2A*<br>*ZNF408*	ARRP	c.2276G>T; p.(Cys759Phe)<br>c.1534dup; p.(Arg512Profs*27)	NP	P1	NA	[1]<br>This study
RP-903	FRP-57	*USH2A*	USH	c.5039A>G; p.(Lys1680Arg)	NP	P2	NA	[5]
RP-904	FRP-58	*USH2A*	USH	c.9799T>C; p.(Cys3267Arg)	SP	NP	NA	[6]
RP-925	-	*USH2A*	USH	c.2276G>T; p.(Cys759Phe)	SP	NP	NA	[1]
RP-963	FRP-140	*USH2A*	USH	c.2276G>T; p.(Cys759Phe)	SP	NP	NA	[1]
RP-1036	FRP-163	*USH2A*	USH	c.2809+1G>A; p.(?)	NP	P1	CC	[7]
RP-1222	FRP-228	*USH1C*	USH	c.1234G>A; p.(Asp412Asn)	NP	P1	NA	[4]
RP-1360	FRP-283	*USH2A*	USH	c.2276G>T; p.(Cys759Phe)	SP	P1	NA	[1]
RP-1387	FRP-320	*PCDH15*	USH	c.3817C>A; p.(Arg1273Ser)	NP	P1	NA	This study
RP-1455	FRP-337	*USH2A*	USH	c.5666A>G; p.(Asp1889Gly)	SP	P1	NA	[8]
**RP-1472**	**FRP-341**	***USH2A***	**USH**	**c.2299del; p.(Glu767Serfs*21)**	**SP**	**NP**	**NA**	[9]
**RP-1485**	**FRP-344**	***USH2A***	**USH**	**c.1214del; p.(Asn405Ilefs*3)**	**SP**	**NP**	**NA**	[10]
**RP-1494**	**FRP-348**	***USH2A***	**USH**	**c.2299del; p.(Glu767Serfs*21)**	**SP**	**NP**	**CC**	[9]
RP-1496	FRP-350	*ADGRV1*	USH	c.3443G>A; p.(Gly1148Asp)	NP	P1	NA	[11]
RP-1541	FRP-367	*USH2A*	USH	c.12067-2A>G; p.?	SP	NP	NA	[12]
**RP-1569**	**FRP-380**	***USH2A***	**USH**	**c.2299del; p.(Glu767Serfs*21)**	**SP**	**NP**	**NA**	[9]
**RP-1595**<br>**RP-1596**	**FRP-389**	***USH2A***	**USH**	**c.10636G>A; p.(Gly3546Arg)**<br>**c.10636G>A; p.(Gly3546Arg)**	**SP**<br>**SP**	**NP**<br>**NP**	**NA**<br>**NA**	[13]
RP-1600	FRP-391	*USH2A*	USH	c.1144G>A; p.(Gln5796*)	SP	P1	NA	[13]
RP-1634	FRP-410	*ADGRV1*	USH	c.17386C>T; p.(Gln5796*)	NP	P1	NA	[14]
RP-1635	FRP-411	*USH2A*	USH	c.2276G>T; p.(Cys759Phe)	SP	NP	NA	[1]
**RP-1647**	**FRP-417**	***USH2A***	**ARRP**	**c.2276G>T; p.(Cys759Phe)**	**SP**	**NP**	**NA**	[1]
RP-1716	FRP-437	*USH2A*	USH	c.5858C>G; p.(Ala1953Gly)	SP	P1	NA	[15]
RP-1726	FRP-448	*USH2A*	USH	c.13514A>G; p.(Tyr4505Cys)	SP	NP	NA	[16]
RP-1727	FRP-449	*USH2A*	USH	c.1214del; p.(Asn405Ilefs*3)	SP	NP	NA	[10]
RP-1728	FRP-446	*USH2A*	USH	c.13514A>G; p.(Tyr4505Cys)	SP	NP	NA	[16]
RP-1736	FRP-455	*USH2A*	USH	c.2299del; p.(Glu767Serfs*21)	SP	NP	NA	[9]
**RP-1741#**	**FRP-460**	***USH2A***	**USH**	**c.7595-2144A>G; p.(Lys2532Thrfs*56)**	**SP**	**P1**	**NA**	[3]
**RP-1776**	**FRP-484**	***USH2A***	**ARRP + SNHL**	**c.2276G>T; p.(Cys759Phe)**	**SP**	**NP**	**NA**	[1]
RP-1815	FRP-505	*USH2A*	USH	c.908G>A; p.(Arg303His)	NP	P2	CC	[17]
RP-1943	FRP-566	*USH2A*	ARRP	c.2276G>T; p.(Cys759Phe)	SP	P1	NA	[1]
RP-1950	FRP-568	*USH2A*	USH	c.2299del; p.(Glu767Serfs*21)	SP	P1	NA	[9]
RP-1953	FRP-570	*USH1C*	USH	c.1859G>T; p.(Arg620Leu)	NP	P1	NA	[18]
RP-1994	FRP-585	*USH2A*	ARRP	c.2299del; p.(Glu767Serfs*21)	SP	P1	NA	[9]

RP-2034	FRP-614	*USH2A*	USH	c.2299del; p.(Glu767Serfs*21)	SP	P1	CC	[9]
**RP-2055**	**FRP-619**	***USH2A***	**USH**	**c.8435_8438del; p.(Thr2812Metfs*17)**	**SP**	**NP**	**NA**	[19]
RP-2106	FRP-645	*USH2A*	USH	c.5933C>T; p.(Pro1978Leu)	SP	NP	NA	This study
RP-2131	FRP-667	*MYO7A*	USH	c.3G>A; p.(?)	NP	P1	NA	[4]
RP-2140	FRP-676	*CDH23*	USH	c.6393del; p.(Ile2132Serfs*11)	NP	P1	NA	[20]
RP-2159	FRP-675	*USH2A*	USH	c.3637_3642dup; p.(Phe1213_Ala1214dup)	SP	P1	NA	This study
RP-2213	FRP-719	*MYO7A*	ARRP	c.640G>A; p.(Gly214Arg)	NP	P1	NA	[21]
RP-2214	FRP-720	*USH2A*	ARRP	c.2431_2432del; p.(Lys811Aspfs*11)	NP	P1	NA	[6]
**RP-2224**	**FRP-722**	***USH2A***	**USH**	**c.2431_2432del; p.(Lys811Aspfs*11)**	**SP**	**NP**	**NA**	[6]
RP-2232	FRP-722	*USH2A*	USH	c.1898C>A; p.(Ser633*)	SP	P1	NA	[7]
RP-2237	FRP-724	*USH2A*	ARRP	c.12575G>A; p.(Arg4192His)	SP	NP	NA	[22]
RP-2238	FRP-725	*USH2A*<br>*PDE6H*	ARRP	c.1724G>A; p.(Cys575Tyr)<br>c.134+2T>C; p.(?)	SP	NP	NA	[23]<br>This study
RP-2239	FRP-726	*USH2A*	ARRP	c.7061G>A; p.(Arg2354His)	SP	P1	NA	This study
RP-2241	FRP-728	*USH2A*	ARRP	c.2276G>T; p.(Cys759Phe)	SP	NP	NA	[1]
RP-2262	FRP-730	*ADGRV1*	USH	c.1875G>A; p.(Asp6252Asn)	NP	P1	NA	This study
RP-2264	FRP-732	*USH2A*	USH	c.1055C>T; p.(Thr352Ile)	SP	P2	NA	[24]
RP-2266	FRP-734	*USH2A*	USH	c.9799T>C; p.(Cys3267Arg)	SP	NP	NA	[6]
RP-2268	FRP-736	*USH2A*	ARRP	c.10073G>A; p.(Cys3358Tyr)	NP	P2	NA	[15]
RP-2269	FRP-737	*USH2A*	ARRP	c.1214del; p.(Asn405Ilefs*3)	NP	P2	NA	[10]
RP-2270	FRP-738	*USH2A*	ARRP	c.12574C>T; p.(Arg4192Cys)	NP	P2	NA	[25]
RP-2275	FRP-740	*USH2A*	USH	c.5776+1G>A; p.(?)	SP	P1	NA	[26]

Homozygous and heterozygous patients for deep-intronic variants in USH2A are highlighted in bold. RP/RPN: Patient number; "-": Unkown; RP/FRPN: Family number; Dx: Diagnosis; ARRP: Autosomal Recessive Retinitis Pigmentosa; USH: Usher Syndrome; SS: Sanger Screening; SP: Screening Performed; NP: Not Performed; P1: Panel design one; P2: Panel design two; SEG: Segregation analysis. The "#" symbol refers to the NGS positive control; CC: Cosegregation confirmed.

Table S2. Sequence of antisense oligonucleotides tested for the aberrant splicing modulation caused by three deep-intronic variants.

Variant	Oligonucleotide	Sequence (3'-5')	G-C%
USH2A: c.14134-3169A>G	AON1-PE64	CCAUUUUUCUAGGAGAAGCCC	47.6
	AON2-PE64	CAACUGUUUGUUCACAAGGU	40
USH2A: c.9958+3438A>G	AON1-PE50	UGCAGGUGUAAAAAUGGCCAAG	45.5
	AON7-PE50	UACCUUGACUGGAGUGAUCUC	47.6
USH2A: c.8681+3960A>G	AON1-PE43	CAGUGACAAUUUGGAUGGAGAC	45.5

G-C: Guanine-Cytosine content.

Table S3. Primer sequences used for the minigene assays

Variant	Restriction enzyme	Rectriction site and tails	Primer sequence (5'-3')
USH2A: c.4106C>T	XhoI	AAGAATCTCGAG	gggaaatccagtacatcacc
	NheI	AAGAATGCTAGC	caatatcaactgagtgtggac
USH2A: c.5168-26A>C	XhoI	AAGAATCTCGAG	cactgcctcctatatttacag
	NheI	AAGAATGCTAGC	catctgaaaggagatggaaac
USH2A: c.9958+3438A>G	XhoI	AAGAATCTCGAG	cctgtactatgactaaccag
	NheI	AAGAATGCTAGC	ggtgttctgacttctcttc
USH2A: c.8681+3960A>G	NdeI	AAGAATCATATG	gaagctgccgaagtgagtc
	NheI	AAGAATGCTAGC	gaaacacgtgtcatggaactg

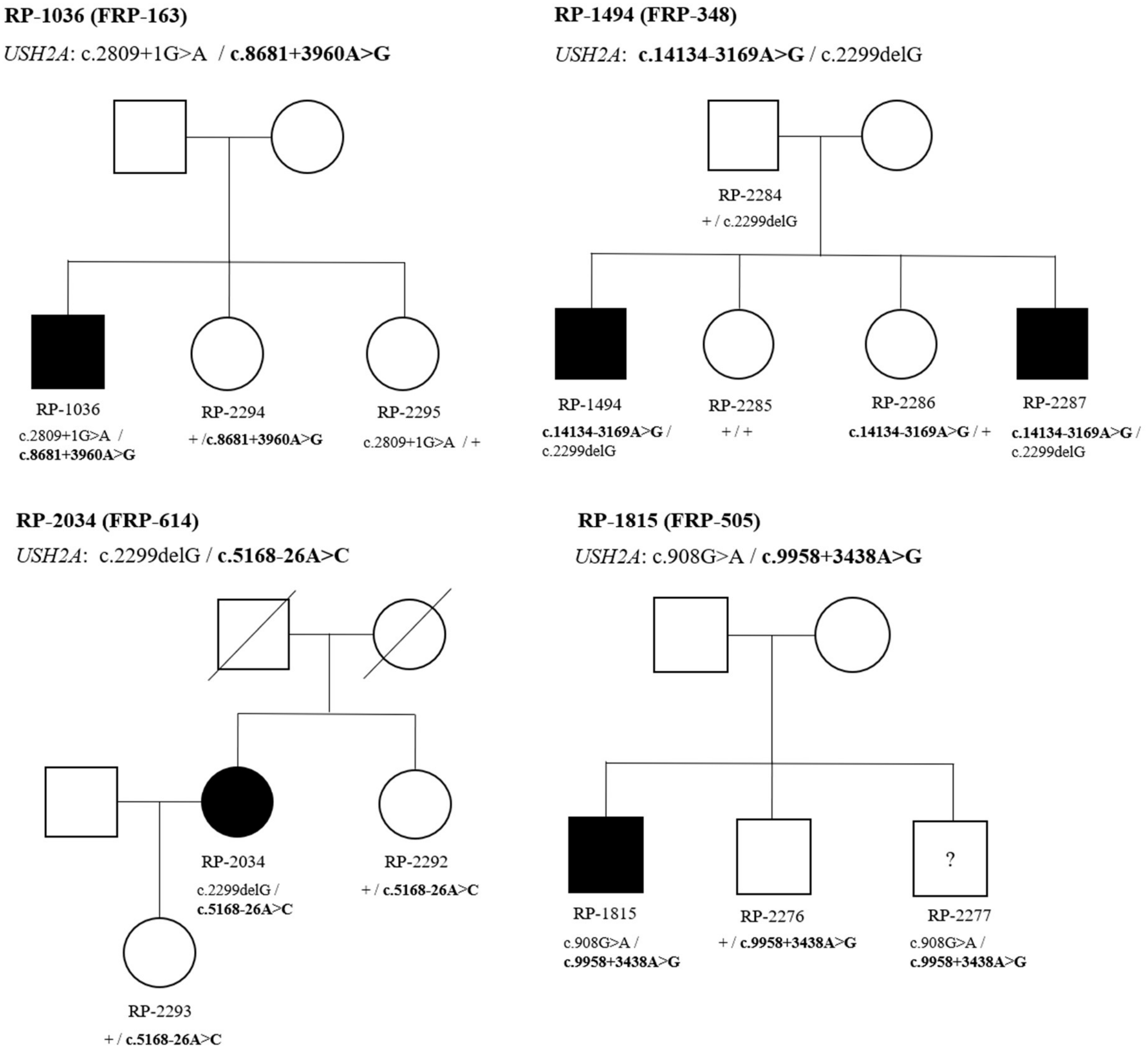

Figure S1. Family pedigrees in which segregation analysis were assessed. Variants identified in this study are highlighted in bold.

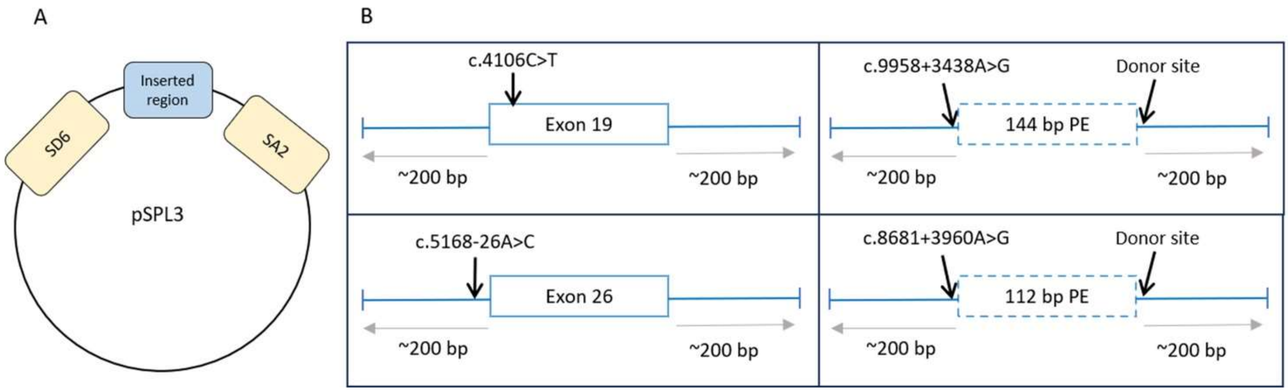

Figure S2. Minigene constructs for the analysis of *USH2A* variants. Regions inserted are depicted in blue in both sections A and B. In section A, constitutive exons are represented in yellow (SD6 and SA2). Each square in section B represents designs performed for each variant. PE: Pseudoexon; bp: base pair.

SUPPLEMENTAL REFERENCES

1. Dreyer, B., Tranebjærg, L., Rosenberg, T., Weston, M.D., Kimberling, W.J., and Nilssen, Ø. (2000). Identification of novel USH2A mutations: implications for the structure of USH2A protein. Eur. J. Hum. Genet. *8*, 500–506. https://doi.org/10.1038/sj.ejhg.5200491.

2. Nishiguchi, K.M., and Rivolta, C. (2012). Genes Associated with Retinitis Pigmentosa and Allied Diseases Are Frequently Mutated in the General Population. PLoS One *7*, e41902. https://doi.org/10.1371/journal.pone.0041902.

3. Vaché, C., Besnard, T., le Berre, P., García-García, G., Baux, D., Larrieu, L., Abadie, C., Blanchet, C., Bolz, H.J., Millan, J., et al. (2012). Usher syndrome type 2 caused by activation of an USH2A pseudoexon: Implications for diagnosis and therapy. Hum. Mutat. *33*, 104–108. https://doi.org/10.1002/humu.21634.

4. Fuster-García, C., García-García, G., Jaijo, T., Fornés, N., Ayuso, C., Fernández-Burriel, M., Sánchez-De la Morena, A., Aller, E., and Millán, J.M. (2018). High-throughput

sequencing for the molecular diagnosis of Usher syndrome reveals 42 novel mutations and consolidates CEP250 as Usher-like disease causative. Sci. Rep. *8*, 17113. https://doi.org/10.1038/s41598-018-35085-0.

5. Costa, K.A., Salles, M.V., Whitebirch, C., Chiang, J., and Sallum, J.M.F. (2017). Gene panel sequencing in Brazilian patients with retinitis pigmentosa. Int. J. Retin. Vitr. *3*, 33. https://doi.org/10.1186/s40942-017-0087-6.

6. Nájera, C., Beneyto, M., Blanca, J., Aller, E., Fontcuberta, A., Millán, J.M., and Ayuso, C. (2002). Mutations in Myosin VIIA (MYO7A) and Usherin (USH2A) in Spanish patients with usher syndrome types I and II, respectively. Hum. Mutat. *20*, 76–77. https://doi.org/10.1002/humu.9042.

7. Baux, D., Blanchet, C., Hamel, C., Meunier, I., Larrieu, L., Faugère, V., Vaché, C., Castorina, P., Puech, B., Bonneau, D., et al. (2014). Enrichment of LOVD-USHbases with 152 USH2A Genotypes Defines an Extensive Mutational Spectrum and Highlights Missense Hotspots. Hum. Mutat. *35*, 1179–1186. https://doi.org/10.1002/humu.22608.

8. Perea-Romero, I., Gordo, G., Iancu, I.F., Del Pozo-Valero, M., Almoguera, B., Blanco-Kelly, F., Carreño, E., Jimenez-Rolando, B., Lopez-Rodriguez, R., Lorda-Sanchez, I., et al. (2021). Genetic landscape of 6089 inherited retinal dystrophies affected cases in Spain and their therapeutic and extended epidemiological implications. Sci. Rep. *11*, 1526. https://doi.org/10.1038/s41598-021-81093-y.

9. Eudy, J.D., Weston, M.D., Yao, S., Hoover, D.M., Rehm, H.L., Ma-Edmonds, M., Yan, D., Ahmad, I., Cheng, J.J., Ayuso, C., et al. (1998). Mutation of a gene encoding a protein with extracellular matrix motifs in Usher syndrome type IIa. Science *280*, 1753–1757.

10. Bernal, S., Medà, C., Solans, T., Ayuso, C., Garcia-Sandoval, B., Valverde, D., Del Rio, E., and Baiget, M. (2005). Clinical and genetic studies in Spanish patients with Usher syndrome type II: description of new mutations and evidence for a lack of genotype-

phenotype correlation. Clin. Genet. *68*, 204–214. https://doi.org/10.1111/j.1399-0004.2005.00481.x.

11. Wang, J., Zhang, V.W., Feng, Y., Tian, X., Li, F.Y., Truong, C., Wang, G., Chiang, P.W., Lewis, R.A., and Wong, L.J.C. (2014). Dependable and efficient clinical utility of target capture-based deep sequencing in molecular diagnosis of retinitis pigmentosa. Investig. Ophthalmol. Vis. Sci. *55*, 6213–6223. https://doi.org/10.1167/iovs.14-14936.

12. Rivolta, C., Sweklo, E.A., Berson, E.L., and Dryja, T.P. (2000). Report Missense Mutation in the USH2A Gene: Association with Recessive Retinitis Pigmentosa without Hearing Loss. Am. J. Hum. Genet *66*, 1975–1978. https://doi.org/10.1086/302926.

13. Garcia-Garcia, G., Aparisi, M.J., Jaijo, T., Rodrigo, R., Leon, A.M., Avila-fernandez, A., Blanco-kelly, F., Bernal, S., Navarro, R., Diaz-llopis, M., et al. (2011). Mutational screening of the USH2A gene in Spanish USH patients reveals 23 novel pathogenic mutations. 1–13.

14. García-García, G., Besnard, T., Baux, D., Vaché, C., Aller, E., Malcolm, S., Claustres, M., Millan, J.M., and Roux, A.-F. (2013). The contribution of GPR98 and DFNB31 genes to a Spanish Usher syndrome type 2 cohort. Mol. Vis. *19*, 367–373.

15. McGee, T.L., Seyedahmadi, B.J., Sweeney, M.O., Dryja, T.P., and Berson, E.L. (2010). Novel mutations in the long isoform of the USH2A gene in patients with Usher syndrome type II or non-syndromic retinitis pigmentosa. J. Med. Genet. *47*, 499–506. https://doi.org/10.1136/jmg.2009.075143.

16. Zampaglione, E., Kinde, B., Place, E.M., Navarro-Gomez, D., Maher, M., Jamshidi, F., Nassiri, S., Mazzone, J.A., Finn, C., Schlegel, D., et al. (2020). Copy-number variation contributes 9% of pathogenicity in the inherited retinal degenerations. Genet. Med. *22*, 1079–1087. https://doi.org/10.1038/s41436-020-0759-8.

17. Yan, D., Ouyang, X., Patterson, D.M., Du, L.L., Jacobson, S.G., and Liu, X.-Z. (2009).

Mutation analysis in the long isoform of USH2A in American patients with Usher Syndrome type II. J. Hum. Genet. *54*, 732–738. https://doi.org/10.1038/jhg.2009.107.

18. Bonnet, C., Riahi, Z., Chantot-bastaraud, S., Smagghe, L., Letexier, M., Marcaillou, C., Lefèvre, G.M., Hardelin, J., El-amraoui, A., Singh-estivalet, A., et al. (2016). An innovative strategy for the molecular diagnosis of Usher syndrome identi fi es causal biallelic mutations in 93 % of European patients. 1730–1738. https://doi.org/10.1038/ejhg.2016.99.

19. Aller, E., Jaijo, T., Beneyto, M., Na, C., Oltra, S., Ayuso, C., Baiget, M., Carballo, M., Antin, G., Navea, A., et al. (2006). Identification of 14 novel mutations in the long isoform of. 1–5. https://doi.org/10.1136/jmg.2006.041764.

20. Oshima, A., Jaijo, T., Aller, E., Millan, J.M., Carney, C., Usami, S., Moller, C., and Kimberling, W.J. (2008). Mutation profile of theCDH23 gene in 56 probands with Usher syndrome type I. Hum. Mutat. *29*, E37–E46. https://doi.org/10.1002/humu.20761.

21. Adato, A., Weiv, D., Kalinski, H., Ayadi, H., Petit, C., and Korostishevsky, M. (1997). Mutation Profile of All 49 Exons of the Human Myosin VIIA Gene , and Haplotype Analysis , in Usher 1 B Families from Diverse Origins. 813–821.

22. Ávila-Fernández, A., Cantalapiedra, D., Aller, E., Vallespín, E., Aguirre-Lambán, J., Blanco-Kelly, F., Corton, M., Riveiro-Álvarez, R., Allikmets, R., Trujillo-Tiebas, M.J., et al. (2010). Mutation analysis of 272 Spanish families affected by autosomal recessive retinitis pigmentosa using a genotyping microarray. Mol. Vis. *16*, 2550–2558. https://doi.org/272 [pii].

23. Bonnet, C., Grati, M., Marlin, S., Levilliers, J., Hardelin, J.P., Parodi, M., Niasme-Grare, M., Zelenika, D., Délépine, M., Feldmann, D., et al. (2011). Complete exon sequencing of all known Usher syndrome genes greatly improves molecular diagnosis. Orphanet J. Rare Dis. *6*, 21. https://doi.org/10.1186/1750-1172-6-21.

24. Baux, D., Larrieu, L., Blanchet, C., Hamel, C., Ben Salah, S., Vielle, A., Gilbert-Dussardier, B., Holder, M., Calvas, P., Philip, N., et al. (2007). Molecular and in silico analyses of the full-length isoform of usherin identify new pathogenic alleles in Usher type II patients. Hum. Mutat. *28*, 781–789. https://doi.org/10.1002/humu.20513.

25. Corton, M., Tatu, S.D., Avila-Fernandez, A., Vallespín, E., Tapias, I., Cantalapiedra, D., Blanco-Kelly, F., Riveiro-Alvarez, R., Bernal, S., García-Sandoval, B., et al. (2013). High frequency of CRB1 mutations as cause of Early-Onset Retinal Dystrophies in the Spanish population. Orphanet J. Rare Dis. *8*. https://doi.org/10.1186/1750-1172-8-20.

26. Sandberg, M.A., Rosner, B., Weigel-DiFranco, C., McGee, T.L., Dryja, T.P., and Berson, E.L. (2008). Disease Course in Patients with Autosomal Recessive Retinitis Pigmentosa due to the USH2A Gene. Investig. Opthalmology Vis. Sci. *49*, 5532. https://doi.org/10.1167/iovs.08-2009.
